# Triggering Receptor Expressed on Myeloid Cells-1 Inhibitor Targeted to Endothelium Decreases Cell Activation

**DOI:** 10.3389/fimmu.2019.02314

**Published:** 2019-10-01

**Authors:** Sébastien Gibot, Lucie Jolly, Jérémie Lemarié, Kevin Carrasco, Marc Derive, Amir Boufenzer

**Affiliations:** ^1^INSERM UMRS-1116, Faculté de Médecine Nancy, Université de Lorraine, Nancy, France; ^2^CHRU Nancy, Hôpital Central, Service de Réanimation Médicale, Nancy, France; ^3^INOTREM SA, Nancy, France

**Keywords:** sepsis, inflammation, TREM-1, endothelium, innate immunity

## Abstract

TREM-1 (Triggering Receptor Expressed on Myeloid cells-1) is an immunoreceptor expressed on neutrophils, monocytes/macrophages, and endothelial cells. It amplifies the inflammatory response driven by Toll-Like Receptors (TLR) engagement. The pharmacological inhibition of TREM-1 confers protection in several pre-clinical models of acute inflammation. In this study, we aimed to investigate the role of TREM-1 in endothelial cells using a sneaking ligand construct (SLC) inhibiting TREM-1 in the endothelium. The SLC was made of 3 modules: an E-selectin targeting domain, a *Pseudomonas aeruginosa* exotoxin a translocation domain, and a 7 aa peptide (LSKSLVF) that contains the interaction site between TREM-1 and its adaptor protein DAP-12. SLC peptide was effectively picked up by endothelial cells following LPS stimulation. It decreased LPS induced TREM-1 up-regulation and cell activation, neutrophils extravasation, and improved median survival time during experimental peritonitis in mice. We reported that a targeted endothelial TREM-1 inhibition is able to dampen cell activation and to confer protection during septic shock in mice. The use of such cell-specific, ligand- independent TREM-1 inhibitors deserve further investigations during acute or chronic inflammatory disorders.

## Introduction

The Triggering Receptor Expressed on Myeloid cells-1 (TREM-1) is an immune-receptor that plays a key role in the amplification of the inflammatory response triggered by Toll-Like Receptors (TLR) or NOD-like receptors (NLR) engagement (1, 2). Membrane TREM-1 presents three distinct domains: an Ig-like structure, a transmembrane part and a cytoplasmic tail, which associates with the adaptor molecule DAP12 (3). TREM-1 has been identified in neutrophils, mature monocytes, macrophages and natural killer cells, and endothelial cells (4–7). To become activated, TREM-1 expression should first be up-regulated following TLR or NLR signaling, then clustered and multimerized at the cell surface. The interaction between TREM-1 and DAP12 is crucial in the stabilization and multimerization of TREM-1 (8). However, even if PGLYRP1 has recently been found to be a good candidate (9), the actual nature of the TREM-1 ligand(s) remains hypothetical. Nevertheless, TREM-1 pharmacological inhibition by the LR12 peptide (that impairs ligand/TREM-1 interaction and blocks the multimerization) or genetic invalidation reduces hyper-responsiveness and death during various experimental septic shock model (10–12), protects from cardiovascular dysfunction following myocardial infarction (13, 14), and prevents atherosclerosis (15, 16), and even cancer (17, 18). This compound has also recently been tested in septic shock patients (19). During this deadly syndrome (20), one of the main organs to become dysfunctional is the endothelium (21). Our group demonstrated that a targeted endothelial *Trem-1* deletion protected mice during septic shock by modulating inflammatory cells mobilization and activation, restoring vasoreactivity, and improving survival (7). Therefore, a specific endothelium-targeted TREM-1 inhibition should be ideal in that it would not alter the capacities of the immune cells in terms of microbial phagocytosis and killing.

Leveraging a new model of transmembrane signaling, the signaling chain homo-oligomerization (SCHOOL) model described by Sigalov et al. (22), we designed a ligand- independent TREM-1 inhibitory peptide that we embedded into a construct that specifically targets the endothelium (23). Here we demonstrated that this peptide was able to reduce endothelial cells TREM-1 expression and activation.

## Materials and Methods

### TREM-1 Sneaking Ligand Construct

**Figure 1:**
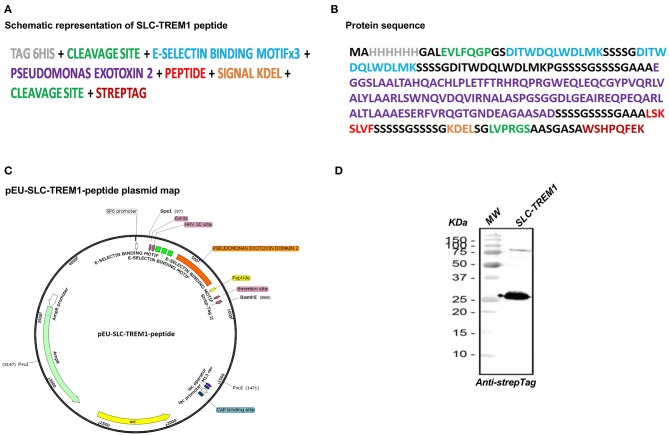
Representation of TREM-1 sneaking ligand construct (SLC). The multimodular synthetic gene is represented in **(A)**, and the corresponding protein sequence in **(B)**. The gene was ligated into the pEU-E01 plasmid **(C)**. Western blot analysis of the recombinant protein revealed by anti-Strep-Tag antibody **(D)**.

SLC-TREM-1 sequence (Figure 1) was subcloned into pEU-E01 plasmid. Plasmid DNA was then transcribed into mRNA with SP6 RNA polymerase that was directly used for translation in a cell-free wheat germ system. The obtained protein was purified by affinity chromatography on a Gravity flow Strep-Tactin Sepharose column (IBA Lifescience, Gottingen, Germany) with a resulting purity >90% and was endotoxin-free. A control SLC-TREM-1 that lacks the E-selectin binding motifs was similarly synthesized.

### Cell Culture and Stimulation

Human pulmonary microvascular endothelial cells (HPMEC) were purchased from Promocell (6 different batches originating from 6 different donors) (Heidelberg, Germany). The cells were maintained in complete endothelial cell growth medium MV (Promocell) at 37°C in a 5% CO_2_ humidified atmosphere incubator. All experiments were performed between passages 2 and 5.

Cells were stimulated in complete medium supplemented with 1 μg/ml *Escherichia coli* LPS (0111:B4; Sigma-Aldrich Saint-Quentin Fallavier, France) in the presence or absence of 250 or 500 nM SLC during various times depending on the experiments. Supernatants were collected for cytokines measurements and cells lysed for protein phosphorylation analyses.

Supernatants from stimulated cells were recovered after 24 h stimulation, and the concentrations of IL-8 and MCP-1 were measured using human Quantikine ELISA kits (R&D Systems, Abingdon, UK) according to the manufacturer's protocol. Cytokine array was performed using the Proteome Profiler kit (R&D Systems).

### Immunoblotting

HPMEC or monocytes were lysed in PhosphoSafe Extraction Reagent (Novagen, Merck Biosciences, Nottingham, U.K.) and centrifuged for 5 min at 16,000 g at 4°C to collect the supernatant. Protein concentration was determined (BCA Protein Assay Kit, Pierce; ThermoScientific), and thirty micrograms of each sample were electrophoresed on a Criterion XT Bis-Tris Gel 4–12% (Bio-Rad) and transferred to a polyvinylidene difluoride membrane (Millipore, Saint-Quentin en Yvelines, France). The membrane was blocked with 5% w/v skim milk powder in TBST (0.1 M Tris-HCl pH 8,1.5 M NaCl and 1% Tween-20) for 2 h at room temperature, and subsequently incubated with anti-TREM-1 (AbD Serotec), anti-(p)ERK1/2, anti-(p)eNOS, anti-(p)P65 (Nuclear Factor-κB p65), and anti-His (Cell Signaling, USA) antibodies overnight at 4°C. After vigorous washing in TBST, the membrane was incubated with a secondary antibody conjugated to horseradish peroxidase for 1h at room temperature. Immunocomplexes were detected with the SuperSignal West Femto Substrate (Pierce; ThermoScientific). Non-phosphorylated forms or tubulin (Cell Signaling) were used for normalization. Acquisition and quantitative signal density analyses were performed by a LAS-4000 imager (FSVT) and Multi-Gauge software (LifeScience Fujifilm, Tokyo, Japan).

### Confocal Microscopy

HPMEC were seeded and stimulated on Nunc LabTek chambers (Thermo Fisher Scientific, Waltham, MA, USA) for 24 h. After stimulation, cells were then washed and fixed with paraformaldehyde (4%) for 20 min, permeabilized with Triton 0.1% for 30 min, and blocked in 1% bovine serum albumin for 1 h prior to incubation, with indicated primary antibodies at 4°C overnight (His, TREM-1, DAP-12) (BIOSS, MA, USA). Nuclei were stained with 1 μg/mL TO-PRO3 (Invitrogen, USA) for 1 h at 37°C. After washing with PBS, coverslips were mounted on Vectashield (Vector Laboratories, CA, USA) and visualized through sequentially scanning on TCS SP5 X confocal microscope (Leica, Wetzlar, Germany). Images were processed using LAS AF Lite blue software (Leica).

### Transmigration Assay

HPMEC were grown to confluency on transwell membranes (6.5-mm diameter and 5-μm pore size; Costar). Cells were then incubated with 10 ng/mL TNF-α in the presence or not of 250 nM SLC at 37°C. After 2 h, 1.5 × 10^6^ neutrophils were added in the upper chamber for 90 min. Migrated neutrophils were recovered from the bottom of the well and counted using an automated cell counter (Biorad, Marnes-la-Coquette, France).

### Animals

Male C57Bl/6 mice were housed in plastic cages maintained on a 12 h light/dark cycle at a controlled temperature (24 ± 2°C) and humidity (50 ± 5%), and allowed free access to standard mice chow and water. Experiments were approved by our Institutional Animal Care and Use Committee (number 01079.01) and conducted according to the guidelines from Directive 2010/63/EU of the European Parliament on the protection of animals used for scientific purposes.

### Thioglycollate-Induced Peritonitis

Thioglycollate-induced peritonitis was induced as previously described (23) and peritoneal neutrophils extravasation was investigated 4 h later. One hundred or two hundred μg of SLC were administered intraperitoneally (i.p.) 30 min after induction.

### Caecal Ligation and Puncture (CLP) Polymicrobial Sepsis Model

Male (6–8 weeks) C57Bl/6 mice were anesthetized with isoflurane. The caecum was exposed through a 1.0 cm abdominal midline incision and subjected to a ligation of the distal half followed by puncture with a G21 needle. A small amount of stool was expelled from the punctures to ensure patency. The caecum was replaced into the peritoneal cavity and the abdominal incision closed in two layers. After surgery, all mice were injected subcutaneously with 0.5 mL 0.9% NaCl solution for fluid resuscitation. Two hours after surgery, animals (*n* = 10 per group) were randomized to receive either 100 μg SLC in 200 μL 0.9% NaCl or 200 μL 0.9% NaCl alone i.p. Survival was next monitored for 10 days.

### Statistical Analysis

All data, unless indicated, were normally distributed and then are presented as mean ± SD. Statistical significance between groups was analyzed using Student *t*-test. Kaplan–Meier survival curves were analyzed using the log-rank test. Statistical analysis was performed using Prism Version 6 software (GraphPad) and a *P* value <0.05 was deemed significant.

## Results

### Construction of a Sneaking Ligand That Inhibits Endothelial TREM-1

We designed the construct based upon the strategy used by Sehnert et al. (23). The SLC is made of 3 modules: (1) a targeting domain consisting of 3 repeats of an E-selectin binding peptide (24) that gives a high avidity for E-selectin [specifically expressed on endothelial cells especially upon stimulation by cytokines or lipopolysaccharide (LPS)]; (2) the translocation domain of *Pseudomonas aeruginosa* exotoxin A to mediate endosomal release in the cytosol (25); (3) and the LSKSLVF peptide that corresponds to the interaction site of TREM-1 (TREM-1_215−221_) with its adaptor protein DAP-12 (3, 26). This region is conserved between species with a 100% homology between human and mouse sequence. Figure 1 shows a schematic representation of the synthetic gene, the corresponding protein sequence, and the plasmid map. Western blot analysis using an antibody against the N-terminal Strep-Tag revealed the high purity of the recombinant protein at the expected molecular weight (Figure 1D).

### SLC-TREM-1 Is Picked Up by Endothelium and Decreases TREM-1 Expression

**Figure 2:**
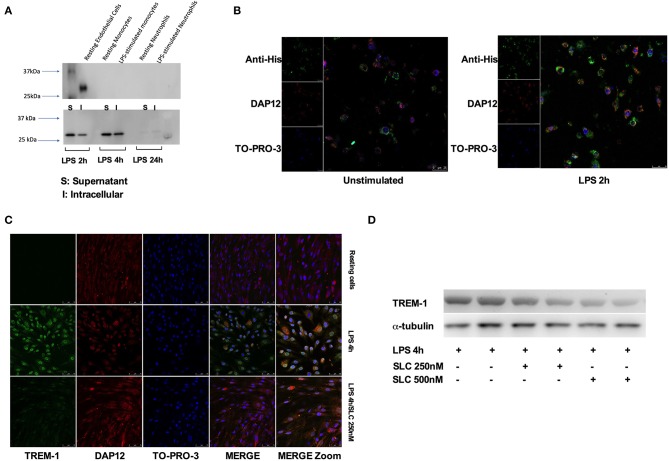
SLC-TREM-1 enters into endothelial cells and decreases TREM-1 expression. **(A)** Western blot analysis of SLC-TREM-1 revealed by anti-His antibody: SLC (250 nM) was found into resting endothelial cells but not into resting or LPS-activated monocytes and neutrophils (left panel). Stimulation with LPS increased penetration of the recombinant protein into endothelial cells (right panel). **(B,C)** Confocal microscopy analysis of SLC-TREM-1 (250 nM) in unstimulated and LPS-activated endothelial cells. **(C,D)** SLC-TREM-1 decreased 4 h LPS (100 ng/mL)-induced TREM-1 up-regulation on endothelial cells as assessed by confocal microscopy (SLC 250 nM) **(C)** and Western blot (two different experiments) (SLC 250 or 500 nM) **(D)**.

We first tested the ability of SLC-TREM-1 to be endocytosed. Whereas, resting or LPS-stimulated primary human monocytes or neutrophils were unable to endocytose SLC, the protein was observed into unstimulated HPMEC after 1-h incubation (Figure 2A). LPS is known to strongly up-regulate E-selectin expression by endothelial cells. We therefore expected LPS to promote SLC capture. Indeed, we observed a strong uptake of SLC by HPMEC after LPS stimulation, as demonstrated by the large amount of SLC detected in HPMEC lysates after 2 and 4-h LPS stimulation (Figure 2A). We confirmed these findings by confocal microscopy: although SLC (green color) was already present in unstimulated HPMEC, LPS increased endocytose release (Figure 2B). SLC seemed to co-localize with DAP-12 and to cluster in most of the cells. We next investigated whether SLC could reduce TREM-1 expression. Upon stimulation by LPS, TREM-1 expression is up-regulated at the membrane and stabilized through its interaction with DAP-12 (8). As previously described (7), LPS effectively up-regulated TREM-1 expression on HPMEC, whereas it was almost undetectable on resting cells. SLC incubation was able to dampen LPS-induced TREM-1 up-regulation as assessed by confocal microscopy (SLC 250 nM) (Figure 2C) and Western blot (SLC 250 or 500 nM) (Figure 2D).

### SLC-TREM-1 Dampens LPS-Induced Endothelial Cells Activation

**Figure 3:**
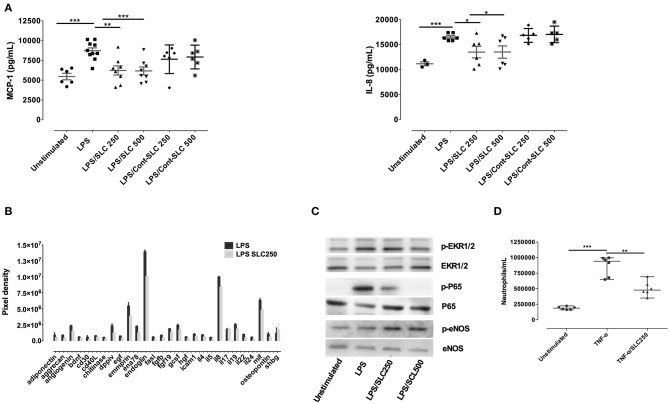
SLC-TREM-1 reduces endothelial cells activation. SLC-TREM-1 decreased endothelial cells cytokines/chemokines production induced by LPS as assessed by ELISA **(A)** or cytokines array **(B)** (two different experiments). It reduced LPS-induced ERK1/2 and p65 phosphorylation while increased p-eNOS **(C)**. SLC-TREM-1 also decreased neutrophils transmigration across TNF-α activated endothelial cells **(D)**. ^*^p < 0.05, ^**^p < 0.01, and ^***^p < 0.001 vs. activated cells.

We next examined the ability of SLC to reduce endothelial cells activation. Following LPS stimulation for 24-h, HPMEC released high amounts of MCP-1 and IL-8 as assessed by ELISA (Figure 3A). SLC reduced the production of these proteins, whichever the concentration used. Cytokine array analysis also showed that SLC was able to decrease numerous cyto/chemokines (Figure 3B), including EMMPRIN or endoglin, markers of endothelial cells activation. An analysis of protein expression in HPMEC by Western blot revealed an increased phosphorylation of ERK1/2 and p65 upon LPS stimulation that was reduced by SLC (Figure 3C). Interestingly, the expression of the constitutive p-eNOS expression, important for endothelial cell fitness, was not reduced.

The interaction of leukocytes with endothelium is increased upon endothelial cells activation. We, therefore, wondered whether SLC could alter leukocytes migration across TNF-α activated endothelium. HPMEC were grown to confluence on transwell membranes and were then incubated with 10 ng/mL TNF-α for 2 h before 1.5 × 10^6^ neutrophils were added in the upper chamber for 90 min. Whereas, TNF-α facilitates neutrophils transmigration, SLC largely reduced this phenomenon (Figure 3D).

### SLC-TREM-1 Effect During Experimental Peritonitis

**Figure 4:**
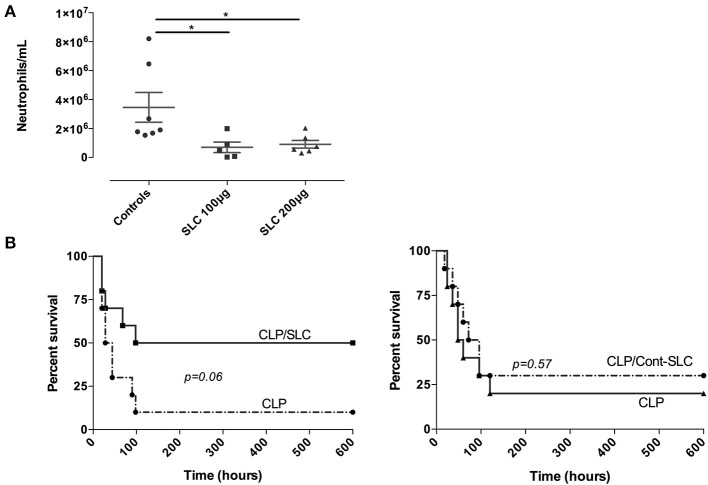
**(A)** Effect of SLC-TREM-1 in experimental peritonitis. Neutrophils count in the peritoneal fluid 4 h after i.p. thioglycolate injection. ^*^p < 0.05 vs. control animals (thioglycolate without peptide), *n* = 5–7 mice per group. **(B)** Kaplan-Meier survival curves after CLP in mice (*n* = 10 per group). Animals randomly received 100 μg SLC-TREM-1 or Cont-SLC in 200 μL NaCl 0.9% or 200 μL NaCl 0.9% i.p 2 h after the onset of CLP.

To investigate if SLC could alter leukocyte trafficking *in vivo*, we performed an acute model of thioglycollate-induced peritonitis in mice. As shown in Figure 4A, the neutrophil influx into the peritoneal cavity was largely reduced by the administration of 100 or 200 μg SLC, suggesting a reduction of vascular permeability.

Finally, we investigated the effect of SLC during a polymicrobial model of peritonitis induced by CLP. We used a single i.p. administration of 100 μg SLC 2 h after the surgery and followed animals for 10 days. In the control group, a 10% survival rate was observed, as compared to 50% in the SLC-treated group, although this difference did not reach statistical significance (*p* = 0.06; Figure 4B). The median survival time was higher in the treated group as compared to the control animals (169 vs. 36 h, *p* < 0.01).

## Discussion

Endothelium dysfunction is the hallmark of septic shock and is responsible for vascular hyporeactivity and activated coagulation, that lead to organ failure and finally death (21, 27). Septic shock is defined by a life-threatening dysregulated host response to infection. Among the mediators involved in this exacerbated immune response, TREM-1 plays a key role (28). During various models of sepsis, we observed that the TREM-1 inhibition, in addition to reducing inflammation and improving survival, prevented from hemodynamic instability (10, 11).

Following the initial paper of Laskin et al. who were the first to suggest a TREM-1 expression in mouse liver ECs, which was upregulated upon stimulation with LPS, TNFα, or IL-1β (29, 30), we have recently shown that a targeted endothelial *Trem-1* deletion protected mice during septic shock (7). Based on robust pre-clinical data, a TREM-1 inhibitory peptide (LR12 or nangibotide) is under clinical development and has just successfully passed a phase 2a trial (19). However, a potential concern that may arise for the broad untargeted inhibition of TREM-1 with LR12 is that it could compromise bacterial clearance by myeloid cells. As such, the design of endothelium-specific TREM-1 inhibitors may prove interesting in preventing endothelial dysfunction while preserving myeloid cells functionality.

We and others (8, 22) have, at least partially, deciphered the mechanics of TREM-1 activation. The expression of this receptor first needs to be up-regulated (following a TLR or NLR engagement), then clustered and multimerized at the cell surface. The interaction between the short cytoplasmic tail of TREM-1 with the ITAM motif of DAP-12 stabilizes both TREM-1 expression and multimerization (8). We thus designed a 7aa peptide previously shown to inhibit TREM-1/DAP-12 interaction (22). As we aimed to specifically address the endothelium, we embedded this peptide into a previously described construct (23) made of a targeting domain consisting of 3 repeats of an E-selectin binding peptide, which gives a high avidity for E-selectin that is specifically expressed on endothelial cells, and the translocation domain of *Pseudomonas aeruginosa* exotoxin A to mediate endosomal release in the cytosol.

We first observed that this TREM-1 “sneaking ligand peptide” was effectively endocytosed by endothelial cells, especially after LPS stimulation, but not by monocytes. In most cells, the SLC-TREM-1 seemed to co-localize with DAP-12. LPS stimulation up-regulated TREM-1 expression on endothelial cells and incubation with SLC-TREM-1 was able to dampen this LPS-induced TREM-1 up-regulation. We also found that SLC-TREM-1 decreased endothelial cells activation. Exaggerated vascular permeability and leukocytes diapedesis across the endothelium is classic during inflammatory processes. We observed that SLC-TREM-1 reduced the migration of neutrophils through the endothelial layer both *in vitro* and during thioglycollate-induced peritonitis in mice. Finally, we wondered whether the administration of SLC-TREM-1 in septic animals could improve survival. Although not statistically significant due to a lack of power (*p* = 0.06), there was a trend toward a survival improvement in animals treated with SLC-TREM-1 (50 vs. 10% in the control group). The median survival time was higher in the treated group as compared to the control animals (169 vs. 36 h, *p* < 0.01).

A limitation of this study is that we did not test the ability of SLC-TREM-1 to be ingested by lymphocytes or epithelial cells. However, the expression of TREM-1 has not clearly been reported on these cells. Moreover, as our study was preliminary, we did not perform an extensive analysis of organ damage or immune cells function leukocytes' trafficking during our experimental septic shock model but monitored survival only. The precise effect of SLC-TREM-1 needs to be further investigated on these readouts, as well as on the bacterial clearance. Indeed, as SLC-TREM-1 affects neutrophil recruitment in our thioglycolate-induced peritonitis, it could compromise effective phagocytosis during the CLP model, although the survival results argue against such a phenomenon.

## Conclusion

We here reported that a TREM-1 inhibitory peptide targeting the endothelium was able to reduce endothelial cell activation and to confer protection during experimental peritonitis in mice. Targeting mechanisms responsible for endothelial dysfunction that occurs during acute inflammatory disorders could be a great interest in the management of critically ill patients.

## Data Availability Statement

The datasets generated for this study are available on request to the corresponding author.

## Ethics Statement

Experiments were approved by our Institutional Animal Care and Use Committee [Comité d'Éthique Lorrain en Matière d'Expérimentation Animale (CELMEA - CE2A-66; approval number 01079.01] and conducted according to the guidelines from Directive 2010/63/EU of the European Parliament on the protection of animals used for scientific purposes.

## Author Contributions

SG and AB designed the study, performed experiments, analyzed data, and wrote the manuscript. LJ, JL, KC, and MD performed experiments and analyzed data. All authors approved the final version of the manuscript.

### Conflict of Interest

MD and SG are co-founders of INOTREM SA, a company developing TREM-1 inhibitors. LJ, KC, and AB are employees of INOTREM. The remaining author declares that the research was conducted in the absence of any commercial or financial relationships that could be construed as a potential conflict of interest.

## Abbreviations

CLP: caecal ligation and puncture
HPMEC: human pulmonary microvascular endothelial cells
LPS: lipopolysaccharide
NLR: NOD-like receptor
SLC: sneaking ligand construct
TLR: Toll-like receptor
TREM-1: triggering receptor expressed on myeloid cells-1
sTREM-1: soluble TREM-1.
